# The Prince's Power for Good

**Published:** 1898-12-24

**Authors:** 


### THE PRINCE'S POWER FOR GOOD.

At the meeting held at Marlborough House in support of the National Association for the Prevention of Consumption, Sir William Broadbent, addressing H.R.H. the Prince of Wales, said: "In regard to the subject of tuberculosis, it is a moderate estimate that at least 200 new persons in Great Britain and Ireland must catch the disease every day. It is the realisation of this fact which has called into existence the National Association for the Prevention of Consumption and other forms of Tuberculosis. The mission of the association is to carry into every dwelling in the and an elementary knowledge of the modes in which consumption is propagated, and of the means by which its spread may be prevented, and thus to strengthen the hands of medical men throughout the country who are dealing with individual cases of this disease. To this end the public attention must be captured, the public imagination must be impressed, the defensive instincts of the general public must be aroused, and we do not hesitate to express our belief that your Royal Highness will, by the single gracious act of calling together and presiding over this meeting, save thousands of lives."

